# Priorities of patients, caregivers and health‐care professionals for health research – A systematic review

**DOI:** 10.1111/hex.13090

**Published:** 2020-07-09

**Authors:** Michael Levelink, Mona Voigt‐Barbarowicz, Anna Levke Brütt

**Affiliations:** ^1^ Department of Health Services Research School of Medicine and Health Sciences Carl von Ossietzky University Oldenburg Oldenburg Germany

**Keywords:** patient and public involvement, prioritization, research agenda, research priorities, systematic review

## Abstract

**Background:**

Based on subjective experience, patients can identify research priorities important for health services research. A systematic method for priority setting has been developed by the James Lind Alliance.

**Objective:**

This article reviews the literature on the research priorities of patients, caregivers and health‐care professionals and presents the prioritized research themes and prioritization methods used.

**Search strategy:**

Three electronic databases were searched on 22 May 2018. The search was not limited to any time period or language.

**Inclusion criteria:**

The included studies reported the identification and prioritization of research priorities involving patients, relatives and caregivers. Each included paper addressed a specific ICD‐coded health problem, and at least one‐third of the sample involved in the prioritization process was affected by the health problem.

**Data extraction and synthesis:**

The 10 top‐ranked research priorities were included in the thematic analysis. With an inductive approach, a system of identified themes and subthemes was developed from the research priorities. Each research priority was assigned to one research theme.

**Main results:**

The priority lists of 34 publications involving 331 research priorities were included. Nine main themes represent the content of the research priorities. The most frequently represented main themes are ‘Treatment’, ‘Patients’ and ‘Health condition’. The distribution of the research priorities varied depending on the health conditions and prioritization methods.

**Discussion and conclusions:**

This review provides a comprehensive overview of the overarching research themes in research priorities of affected individuals. The results can guide future patient‐oriented research.

## 1 INTRODUCTION

Currently, a paradigm shift is occurring in health care and related research towards the needs of individuals affected by diseases.^1^ So that their needs are recognized, the people affected by diseases are increasingly involved in different stages of the research process and acknowledged as experts with valuable‐specific disease experience.^2^, ^3^, ^4^ On the one hand, this development stems from the idea that individuals affected by diseases ultimately bear the burden of the diseases and therefore have the right to participate in the process of determining the direction of affiliated research.^5^ On the other hand, if research addresses genuine problems in health care more adequately through patient involvement, scientific findings are more likely to be implemented into practice, thus leading to an efficient use of limited funding resources.^6^ Developing research agendas are a key strategic factor in this context when they are used for the conception of funding programmes and research projects, as they determine the content alignment of future research. This concept applies in particular to evidence‐based medicine since research is a prerequisite for the evidence and thus for the practical use of an intervention.^7^ To date, the research priorities for health research have been predominantly determined by researchers and funding bodies. These research priorities are identified from a scientific or commercial point of view, and the needs of the affected people are not necessarily considered.^8^ However, according to the outlined paradigm shift, an increasing number of initiatives are being developed to include patients, caregivers and health‐care professionals (HCPs) in the process of identifying research priorities.^9^ These research agendas have already been addressed in national research funding programmes.^10^

The inclusion of patients in the process of identifying research priorities has been explicitly discussed since the 1990s. An important milestone of this development is the establishment of the James Lind Alliance (JLA) in 2004. The JLA aims to identify and prioritize unanswered research priorities for specific health conditions with affected patients, caregivers and practitioners. In addition, the initiative uses extensive networking to ensure that the research agendas that are developed are actually being pursued.^11^ The JLA facilitates a specific method in which research agendas are developed by disease‐specific priority setting partnerships (PSP); different stakeholders are equally involved in all stages of the prioritization process. The course of a PSP can be described in different stages. From the results, an interim shortlist of research priorities is developed. It is used as the starting position for the third stage of a PSP, where a prioritization workshop is held to develop a ranked top 10 list of disease‐specific research priorities.^12^ From the results, an interim shortlist of research priorities is developed. It is the basis for the third stage of a PSP, when a prioritization workshop is held. By using an adapted nominal group technique, a ranked top 10 list of disease‐specific research priorities is developed. The guidebook presents the methodological basis of the JLA approach. It has been continuously revised in recent years and is now in its eighth version.^13^ The JLA approach can be considered well established and is supported by research infrastructure in the United Kingdom.^14^ In addition, there are further methods that have been used to identify and prioritize research priorities together with patients or the public, such as focus groups, voting exercises or citizen juries.^15^ The structured JLA approach offers transparency and replicability, but also requires resources and supervision. Further methods can easily be tailored for specific groups or settings.^14^

Overall, the increase in efforts to improve the quality of health care and health research by involving the affected people in the process of identifying research priorities can be assessed: a comprehensive review of this development through 2008 has been provided by Stewart and colleagues. They identified 27 papers in which patients were actively involved in the identification and prioritization of research priorities. Moreover, their analysis reported a broad variety of disease‐related health topics for which patients and clinicians identified research priorities. However, neither the extent of lay involvement nor the contents of the research priorities were analysed.^9^ Accordingly, the work did not provide any conclusions about which research priorities were identified by the affected individuals. Crowe and colleagues approached this question in an analysis of 14 PSPs based on the JLA methodology. Their review focused on research priorities and how they are addressed in on‐going research. Only about 18% of the research priorities concern drugs, vaccines and biologicals, and 23% radiotherapy, surgery and devices, whereas 60% have been linked to ‘other intervention’ including education and training, service delivery as well as psychological and physical therapies. In contrast, actual research studies mainly focus on drug interventions.^16^

On the basis of this background information, we aim to identify overarching research themes identified in prioritization studies with substantial involvement of patients affected by different health conditions and their caregivers.

## 2 METHODS

We conducted a systematic review guided by the Preferred Reporting Items for Systematic Reviews and Meta‐Analyses (PRISMA) statement ^17^ to ensure transparent and complete reporting (Appendix S1). The protocol is not registered.

### 2.1 Search strategy

The PubMed, SCOPUS and PsycINFO databases were systematically searched on 22 May 2018. The keywords used were as follows: patients, carers, service users, clients, consumers, lay AND priorit* AND research OR James Lind Alliance, James Lind Initiative. The search strategy was adapted for the different databases by applying the respective operators, and it was not limited to any time period or language. The detailed search strategy is provided in Appendix S2.

### 2.2 Study selection

The study selection process was performed in two steps. First, the titles and abstracts of identified articles were screened. Original research papers that reported the prioritization of at least five research priorities involving patients or caregivers were included. When only priorities for a specific type of treatment (eg surgical treatments) were ascertained, the respective study was excluded. Second, full‐text articles were obtained for all studies assessed as eligible in the abstract screening process. For the full‐text screening process, the following inclusion criteria were added: studies that are published in a peer‐reviewed journal, studies that focus on a specific ICD‐coded health problem and studies in which the identification and prioritization processes were distinct phases and finally a ranking of the identified research priorities was carried out. To ensure substantial involvement of the affected people, they had to constitute at least one‐third of the sample that performed the prioritizing task for study inclusion (for details, see Table 1). Due to a lack of quality assessment tools for the prioritization studies, quality assurance aspects were addressed by the selection criteria. Two researchers (ML, MVB) screened the references and full texts. In the case of a discrepancy, a third reviewer was consulted (ALB).

**Table 1:** Inclusion criteria

Inclusion criteria: Full‐text screening	
Study design	
IC 1	Papers report original research.
IC 2	Research topics or questions are prioritized.
IC 3	The research objective relates to a specific ICD‐coded health problem.
IC 4	Papers do not focus on specific treatment aspects.
Population	
IC 5	Sample or separable subsample is affected by the same health problem as a patient or caregiver.
IC 6	The proportion of affected persons in the sample is documented as well as whether they are directly or indirectly affected.
IC 7	Participants are at least 18 years old.
IC 8	Affected people are involved in identification and prioritization of research priorities.
IC 9	The prioritizing sample comprises at least one third affected people.
Intervention	
IC 10	Methodical identification and prioritization of research priorities as well as corresponding analysis.
IC 11	Identification and prioritization take place in two distinct phases.
IC 12	The methodical approach is reproducibly documented.
Outcome	
IC 13	A list of at least five research priorities is provided.
IC 14	Research questions in the lists are ranked involving affected persons.

### 2.3 Data extraction

One author (ML or MVB) extracted information from the papers into a summary table (Table 2), and a second reviewer (ML or MVB) checked the content for accuracy. This information included data on the author, year of publication, health conditions of the participants, country in which the study was conducted, methods (identification, interim prioritization and prioritization) and the number of research priorities.

**Table 2:** Data extraction table

Authors, year	Health condition	Country	Elicitation methods	Interim prioritization methods	Prioritization methods	Number of prioritized research themes
Aliberti et al,^52^ 2016	Bronchiectasis	22 European countries	Systematic review, workshop		Survey, workshop	29
Banfield et al,^51^ 2014	Depression and bipolar disorder	Australia	Focus groups, telephone interviews		Focus groups, telephone interviews	16
Bernges et al,^41^ 2017	Depression	Germany	Literature search, survey		Survey	10
Britton et al,^40^ 2017	Barrett's oesophagus and gastro‐oesophageal reflux disease	United Kingdom	Survey	Survey	Workshop	10
Broerse et al,^50^ 2009	Burn injuries	Netherlands	Literature search, interviews, focus groups	Focus groups	Survey, workshop	15
Caron‐Flintermann et al,^56^ 2005	Asthma and Chronic obstructive pulmonary disease (COPD)	Netherlands	Focus groups, feedback meeting		Survey	15
Corner et al,^48^ 2007	Cancer	United Kingdom	Workshop		Workshop	13
Davila‐Seijo et al,^20^ 2013	Dystrophic Epidermolysis Bullosa	Spain	Literature search, systematic review, survey	Survey	Workshop	10
Deane et al,^35^ 2014	Parkinson's disease	United Kingdom	Systematic review, survey	Survey	Workshop	10
Eleftheriadou et al,^34^ 2011	Vitiligo	United Kingdom	Update systematic review, literature search, survey	Workshop	Workshop	12
Finer et al,^33^ 2018	Type 2 diabetes	United Kingdom	Literature search, survey	Survey	Workshop	10
Franklin et al,^39^ 2017	Aphasia following stroke	United Kingdom, Ireland	Survey	Survey	Workshop	10
Gadsby et al,^32^ 2012	Type 1 diabetes	United Kingdom	Literature search, survey	Workshop	Workshop	10
Hemmelgarn et al,^38^ 2017	Non‐dialysis chronic kidney disease	Canada	Literature search, survey	Workshop	Workshop	10
Herbison et al,^47^ 2009	Women living urinary incontinence	New Zealand	Workshop		Workshop	5
Ingram et al,^31^ 2014	Hidradenitis suppurativa	United Kingdom	Survey		Survey, workshop	10
Jones et al,^57^ 2017	Kidney cancer	Canada	Literature search, survey	Workshop	Workshop	10
Khan et al,^29^ 2017	Hypertension	Canada	Literature search, systematic review, survey	Workshop	Survey	10
Lechelt et al,^28^ 2017	Head and neck cancer	Canada	Survey	Survey	Workshop	10
Manns et al,^27^ 2014	Kidney failure	Canada	Literature search, survey	Workshop	Workshop	10
McAllister et al,^46^ 2012	Mental health	Australia	Focus groups	Focus groups	Focus groups	10
Pollock et al,^26^ 2014	Stroke	United Kingdom	Literature search, survey, stroke groups, meetings	Survey	Workshop	10
Prior et al,^30^ 2017	Miscarriage	United Kingdom	Literature search, survey	Survey	Workshop	10
Rees et al,^37^ 2016	Gestational diabetes mellitus	Canada	Literature search, survey, interviews	Workshop	Workshop	10
Restall et al,^45^ 2016	HIV	Canada	Workshop		Voting	10
Rose et al,^44^ 2008	Mental health	United Kingdom	Workshop		Workshops	14
Rowbotham et al,^25^ 2017	Cystic fibrosis	United Kingdom	Survey	Workshop	Survey, workshop	10
Stephens et al,^24^ 2015	Mesothelioma	United Kingdom	Literature search, survey	Survey	Workshop	13
Thomas et al,^23^ 2017	Cellulitis	United Kingdom	Survey	Survey	Workshop	20
Tong et al,^36^ 2015	Chronic kidney disease (CKD)	Australia	Literature search, focus groups	Focus groups	Focus groups	13
van Merode et al,^43^ 2016	Multiple myeloma or Waldenstrom's disease	Netherlands	Interviews, focus groups		Survey, workshop	10
van Middendorp et al,^22^ 2016	Spinal cord injury	United Kingdom	Systematic reviews, survey	Survey	Workshop	10
Wan et al,^21^2016	Endometrial cancer (EC)	United Kingdom	Literature search, survey	Survey	Workshop	10
Young & Chesson,^42^ 2007	Mild learning disabilities	United Kingdom/Scotland	Interviews, focus groups	Focus groups	Survey	6

### 2.4 Data analysis

For analysis of the priority lists, we used a descriptive thematic synthesis, considering suggestions for incorporating qualitative evidence into systematic reviews.^18^ Researchers (ML, MV) copied all reported research priorities from the included papers into the qualitative data analysis software MAXQDA 2018.^19^ The 10 top‐ranked priorities of each paper were included in the further analysis. If lists displayed less than 10, all priorities were included. For synthesis of the priority lists, we used a descriptive thematic synthesis, considering the outline for the use in systematic reviews by Dixon‐Woods and colleagues.^18^ Using an inductive approach, two authors (ML, MV) independently assigned a code to each of the research priorities according to the coding rules (Appendix S3). After one‐third of the research priorities were coded, the codes were independently conceptualized in main and subthemes. The identified themes and subthemes created by ML and MVB were discussed and merged by the three authors (ML, MVB and ALB). ML and MVB coded the remaining research priorities independently using the merged themes and subthemes. Disagreements were again discussed by all three authors, and the identified themes and subthemes were refined in an iterative process. Finally, themes and subthemes were agreed upon by the authors and applied to the data (Appendix S4). The final themes and subthemes were used as the basis for further analyses.

## 3 RESULTS

### 3.1 Search strategy and studies

**Figure 1:**
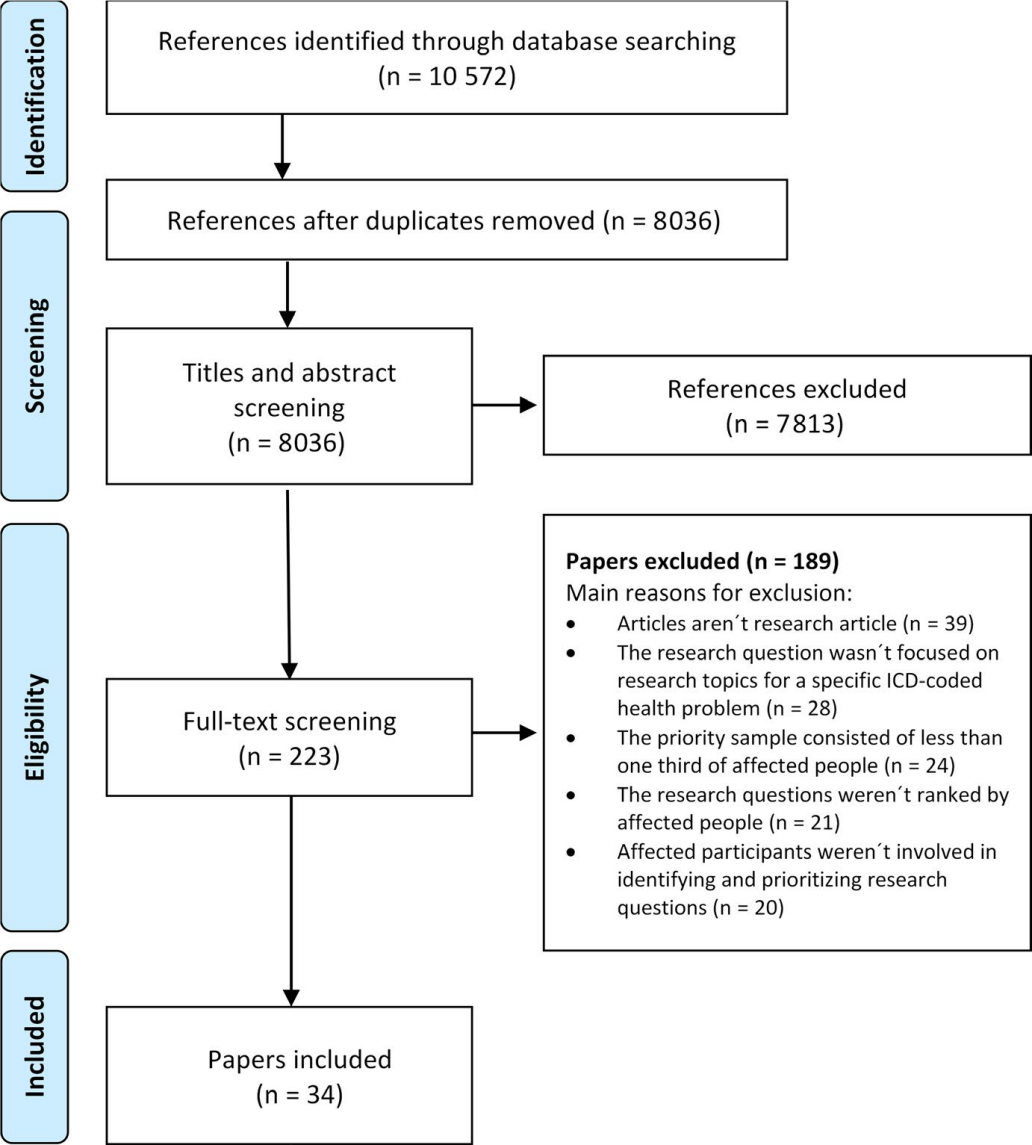
PRISMA 2009 flow diagram

Across all databases, the search yielded 10,572 citations, of which 8,036 remained after the duplicates were removed. After the title and abstract screening process was performed, 223 publications underwent full‐text screening. Of these publications, 34 met the eligibility criteria and were included in the analysis. The most common reasons for exclusion were as follows: articles did not report original research (n = 39), and the study objective did not focus on research priorities for a specific ICD‐coded health problem (n = 28) (Figure 1).

### 3.2 Study characteristics

The articles were published between 2005 and 2018, and half of the articles (n = 17) were published in 2016 or later. All included studies were conducted in Western countries, and most of them were conducted in the UK (n = 17). In our sample, 17 studies were JLA PSPs. This method was adapted in six additional studies without being directly supported by the JLA. The other studies (n = 11) used different methods for identifying research priorities, namely, group discussions, interviews and workshops. The prioritization methods for ranking research priorities included surveys, group discussions, voting exercises and consensus meetings. The diseases that the studies focused on covered a broad range of ICD classifications, including neoplasms C00 ‐ D48 (n = 6; eg head and neck cancer), mental and behavioural disorders F00‐F99 (n = 5; eg depression) and diseases of the genitourinary system N00‐N99 (n = 4; eg chronic kidney disease). Overall, 3538 participants identified research priorities, and this number ranged from 13 to 785 participants per study. Most studies (n = 24) had a generic objective that was used to identify the most important research priorities related to a particular health condition, and 10 studies focused on treatment‐specific uncertainties. The number of research priorities included in the 34 investigated priority lists ranged from 5 to 29 per study, resulting in a total of 398. According to our coding rules (see Appendix S3), the first ten research priorities of each list (n = 331) were included for further analysis.

### 3.3 Summary of the themes in the priority lists

**Figure 2:**
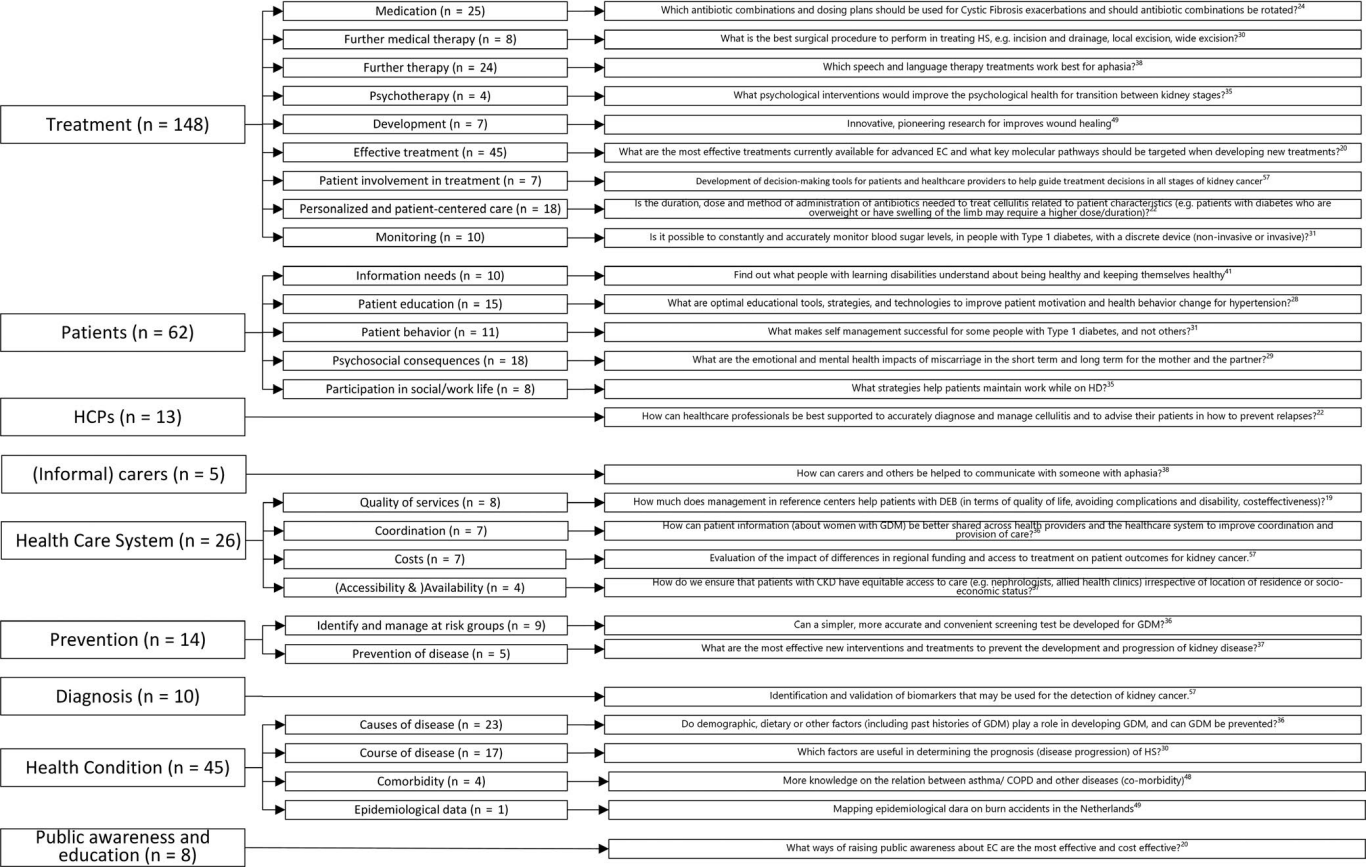
Identified themes and subthemes

The research priorities were subsumed in nine identified main themes with additional subthemes (Figure 2). The identified themes and subthemes summarized the content of the included priority lists and provided an overview of the research priorities that are frequently prioritized by patients, caregivers and health‐care professionals.

### 3.4 Treatment

Nearly half of the research priorities (n = 148) related to the development and evaluation of a disease‐specific treatment (eg effective treatment, personalized and patient‐centred care, medication, therapy forms). These research priorities were summarized in the main theme ‘Treatment’, which is divided into nine subthemes. The research priorities that prompt the identification of best treatment options or a comparison between specific treatment types were subsumed in the research subtheme ‘Effective treatment’ (n = 45). The research priorities related to ‘Medication’ (n = 25) prompted an evaluation of medication regimens, methods of application or medication side‐effects and drug interactions. The subtheme ‘Personalized and patient‐centred care’ (n = 18) included research priorities that take into account patients´ individual needs and preferences regarding health services. The related research priorities prompted research studies on the relevance of patient‐related characteristics and culturally sensitive therapies. Moreover, the research priorities on individual treatment responses were included in this research subtheme. The additional three subthemes referred to different treatments that have to be performed by medical or special therapists. ‘Further medical therapy’ (n = 8) included all the research priorities dealing with different treatments that need to be applied by medical staff, such as the implantation of artificial organs. Another 24 research priorities addressed different treatments offered by therapists (‘Further therapies’) such as physiotherapy or speech therapy. Furthermore, ‘Psychotherapy’ (n = 4) included research priorities concerning psychological interventions and their effectiveness. ‘Monitoring’ (n = 10) included research priorities that relate to the monitoring of health parameters such as vital signs or to the development of strategies for the optimization of routine measurements and follow‐ups. Seven research priorities focused on innovative, pioneering research on the development new, effective treatments and addressed their potential for implementation in health care. These research priorities were subsumed in the subtheme ‘Development’ (n = 7). ‘Patient involvement in treatment’ (n = 7) comprised research priorities that address the possibilities of actively involving patients in their own health care or health services, for example, through shared decision making.

### 3.5 Patients

The research theme ‘Patients’ was addressed in 62 research priorities and divided into five subthemes. The first subtheme included research priorities that focus on the ‘Psychosocial consequences’ (n = 18) of a health condition in the affected individuals and on coping with a disease. The second subtheme comprised research priorities on how to improve ‘Patient education’ (n = 15) and to inform patients about a disease. Another subtheme concerned the ‘Information needs’ (n = 10) of patients regarding the underlying health condition, treatment options or access to research. The subtheme ‘Patient behaviour’ (n = 11) comprised research priorities questions on the impact of patient lifestyles and self‐management issues. Another 8 research priorities addressed the patients’ ‘Participation in social/work life’.

### 3.6 Health‐care professionals (HCPs)

The research theme ‘Health care professionals’ comprised research priorities (n = 13) that focus on HCPs regarding their attitudes, roles, education, communication and effectiveness. For example, these research priorities prompted research studies that investigate whether HCPs need more training on person‐centred skills.

### 3.7 (Informal) Carers

Research priorities in the research theme ‘(Informal) Carers’ (n = 5) addressed carer needs regarding education and emotional support. Specifically, the linked research priorities questions prompted research on family‐involved care and support by family and friends in situations in which the affected person has communication impairments.

### 3.8 Health‐care system

We identified 26 research priorities that relate to the main theme ‘Health care system’. The subtheme ‘Coordination’ (n = 7) subsumed research priorities that address the optimization of organizational functions and coordinative aspects of the health‐care processes within the system. Another seven research priorities that addressed research on financial and societal ‘Costs’ caused by a disease were identified. Another subtheme addressed the ´Accessibility´ (n = 4) of health services used or needed by specific patient groups. These research priorities pertained to equal access to health care.

### 3.9 Prevention

‘Prevention’ (n = 14) is subdivided into two subthemes. The first subtheme comprised research priorities on screening measures (eg for specific diseases such as type 2 diabetes or Barrett's oesophagus) to ‘Identify and manage risk groups’ (n = 9). The second subtheme included research priorities that focus on research on the development of ways to prevent the onset of a disease (‘Prevention of disease’, n = 5).

### 3.10 Diagnosis

The main theme ‘Diagnosis’ was related to research priorities that prompt an earlier or more precise ‘Diagnosis’ (n = 10) of the disease and related symptoms. The research priorities in this research theme focused on the identification of different types of the respective disease or patient groups and on the criteria for the use of different investigation procedures, as well as the value of the clinical investigations.

### 3.11 Health condition

The research priorities classified as ‘Health condition’ (n = 45) aim to enhance knowledge on specific aspects of the health condition that affects the respective sample. The subtheme ‘Causes of disease’ (n = 23) subsumed research priorities that focus on risk factors and causes of the onset of a disease or associated symptoms. Another subtheme comprised research priorities that focus on factors influencing the ‘Course of the disease’ (n = 17) and its predictability using diagnostic procedures. An additional subtheme addressed the possibilities of managing and preventing ‘Comorbidities’ (n = 4). In addition, one research priorities referred to the mapping of more specific ‘Epidemiological data’ (n = 1).

### 3.12 Public awareness and education

Research priorities in the research theme ‘Public Awareness and education’ (n = 8) indicated a need for research that raised public awareness regarding the patients’ situations. Specifically, the linked research priorities prompted research on effective methods of increasing awareness and educational campaigns to improve the situation of the affected individuals and to reduce stigmatization.

#### 3.12.1 Distribution of themes within priority lists

The priority lists varied in their inclusion of different main themes (Table 3). The main theme ‘Treatment’ was the most prevalent theme across the priority lists. It was included in 88% (n = 30) of the investigated lists. ‘Patients’ was the second most prevalent main theme and was reflected in 76% (n = 26) of the lists. ‘Health condition’ was addressed in 62% (n = 21) of the lists.

**Table 3:** Distribution of themes within priority lists

	Methods			Health conditions				Main research themes									Summary	
	JLA	JLA adopted	other methods	Mental disorders	Genitourinary system	Neoplasms	Other diseases	Prevention	Diagnosis	Treatment	(informal) carers	HCPs	Patients	Public awareness and education	Health‐Care System	Health Condition	number of analysed priorities in list	Research themes per list
***Themes per priority list***								**n**									**n**	**n**
Aliberti et al, 2016			x				x	0	0	1	0	1	3	0	1	4	10	5
Banfield et al, 2014			x	x				0	1	3	0	1	3	1	0	1	10	6
Bernges et al, 2017		x		x				0	0	3	0	0	3	1	3	0	10	4
Britton et al, 2017		x					x	1	0	7	0	0	0	0	1	1	10	4
Broerse et al, 2009			x				x	0	0	5	0	0	0	0	1	4	10	3
Caron‐Flinterman et al, 2005			x				x	1	0	3	1	0	1	0	2	2	10	6
Corner et al, 2007			x			x		0	2	3	0	0	2	1	1	1	10	6
Davila‐Seijo et al, 2013	x						x	0	0	9	0	0	0	0	1	0	10	2
Deane et al, 2014	x						x	0	1	9	0	0	0	0	0	0	10	2
Eleftheriadou et al, 2011	x						x	0	0	10	0	0	0	0	0	0	10	1
Finer et al, 2018	x						x	1	0	3	0	0	3	0	0	3	10	4
Franklin et al, 2017		x					x	0	0	5	2	0	2	0	0	1	10	4
Gadsby et al, 2012	x						x	0	0	4	0	1	4	0	0	1	10	4
Hemmelgarn et al, 2017		x			x			2	0	3	0	0	1	0	1	3	10	5
Herbison et al, 2009			x		x			0	0	0	0	0	2	1	1	1	5	4
Ingram et al, 2014	x						x	0	0	6	0	0	1	0	0	3	10	3
Jones et al, 2017b	x					x		0	2	4	0	0	1	0	1	2	10	5
Khan et al, 2017	x						x	0	0	6	0	1	3	0	0	0	10	3
Lechelt et al, 2017	x					x		0	0	5	0	1	2	0	1	1	10	5
Manns et al, 2014	x				x			0	0	4	0	0	1	0	0	5	10	3
McAllister et al, 2012			x	x				0	0	2	1	1	5	0	1	0	10	5
Pollock et al, 2012	x						x	0	0	7	0	0	3	0	0	0	10	2
Prior et al, 2017	x						x	2	2	0	0	0	2	0	0	4	10	4
Rees et al, 2016		x					x	2	0	3	0	0	2	0	1	2	10	5
Restall et al, 2016			x				x	0	0	0	0	0	5	1	4	0	10	3
Rose et al, 2008			x	x				0	0	5	0	1	0	1	2	1	10	5
Rowbotham et al, 2017	x						x	0	0	8	0	0	1	0	0	1	10	3
Stephens et al, 2015	x					x		2	0	8	0	0	0	0	0	0	10	2
Thomas et al, 2017	x						x	1	2	6	0	1	0	0	0	0	10	4
Tong et al, 2015		x			x			0	0	6	0	0	3	1	0	0	10	3
van Merode et al, 2016			x			x		0	0	1	0	2	4	0	3	0	10	4
van Middendorp et al,	x						x	0	0	6	0	0	1	0	1	2	10	4
Wan et al, 2016	x					x		2	0	3	0	0	2	1	0	2	10	5
Young & Chesson, 2007			x	x				0	0	0	1	3	2	0	0	0	6	3
Total	17	6	11	5	4	6	19	14	10	148	5	13	62	8	26	45	331	131
	**JLA**	**JLA adopted**	**other methods**	**Mental disorders**	**Genitourinary system**	**Neoplasms**	**Other diseases**	**Prevention**	**Diagnosis**	**Treatment**	**(informal) carers**	**HCPs**	**Patients**	**Public awareness** **and education**	**Health‐Care System**	**Health Condition**	**Different themes in lists (Mean)**	
								**%**									**M**	
**Total** **(n = 34)**								26	18	88	12	29	76	24	50	62	3.85	
***Themes by methodological approach***																		
JLA (n = 17)	x							29	24	94	0	24	71	6	24	59	3.29	
JLA adopted (n = 6)		x						50	0	100	17	0	83	33	67	67	4.17	
Other methods (n = 11)			x					9	18	73	27	55	82	45	82	64	4.55	
***Themes by disease***																		
Mental disorders (n = 5)				x				0	20	80	40	80	80	60	60	40	4.60	
Genitourinary system (n = 4)					x			25	0	75	0	0	100	50	50	75	3.75	
Neoplasms (n = 6)						x		33	33	100	0	33	83	33	67	67	4.50	
Other diseases (n = 19)							x	32	16	89	11	21	68	5	42	63	3.47	

Abbrevations: HCP, Health‐care Professionals; JLA, James Lind Alliance method.

The average number of main themes included in the priority lists was 3.85 (range: 1‐6). A total of 16 priority lists included more than four research priorities related to the main theme ‘Treatment’. In two priority lists, more than four research priorities addressed the main theme ‘Patients’, and in one priority list, more than four research priorities were assigned to the research theme ‘Health condition’.

#### 3.12.2 Distribution of themes across the methodological approaches

We identified three different methodological approaches. The JLA method was used in 17 studies.^20^, ^21^, ^22^, ^23^, ^24^, ^25^, ^26^, ^27^, ^28^, ^29^, ^30^, ^31^, ^32^, ^33^, ^34^, ^35^ The most prevalent main theme in the JLA priority lists was ‘Treatment’, which was included in 94% (n = 16) of the JLA priority lists. The main themes ‘Patients’ (71% (n = 12)) and ‘Health condition’ (59% (n = 10)) were also frequently represented. The average number of main themes included in the JLA priority lists was 3.29, with a range from 1 to 5 main themes per list. At least five research priorities regarding the main theme ‘Treatment’ were included in 11 JLA priority lists. More than four research priorities related to the main theme ‘Health condition’ were included in one priority list.

Another method was the adaptation of the mentioned JLA method in six studies.^36^, ^37^, ^38^, ^39^, ^40^, ^41^ These studies used the JLA method but were not supported by the JLA. The main themes ‘Treatment’ (100%, n = 6), ‘Patients’ (83%, n = 5) and ‘Health care system’ (67%, n = 4) were most frequently included in the related priority lists. The average number of main themes included in the priority lists of studies using an adapted JLA method is 4.17, and it ranged from 3 to 5. More than four research priorities related to the main theme ‘Treatment’ were mentioned in three priority lists. For all other main themes, fewer than five research priorities were mentioned.

Furthermore, eleven studies^42^, ^43^, ^44^, ^45^, ^46^, ^47^, ^48^, ^49^, ^50^, ^51^, ^52^ used different methods to identify (eg interviews) and prioritize (eg dotmocracy voting) research priorities. The main themes ‘Patients’ and ‘Health care system’ were most prevalent in these priority lists and were included in 82% (n = 9) of the lists. The second most prevalent main theme was ‘Treatment’, which was reflected in 73% (n = 8) of the priority lists. The average number of main themes included in the priority lists of studies using different methods was 4.55, with a range from 3 to 6 themes per list. More than four research priorities in two lists related to the main themes ‘Treatment’ and ‘Patients’.

#### 3.12.3 Distribution of research themes across health conditions

When the priority lists were contrasted according to the health condition that affected the sample, different focus areas were identified. We investigated three different groups of health conditions that varied in the inclusion of main research themes.

Six studies dealt with research priorities for neoplasms (C00‐D48).^21^, ^24^, ^28^, ^30^, ^43^, ^48^ The main research theme ‘Treatment’ was included in 100% (n = 6) of these priority lists. ‘Patients’ was the second most prevalent main research theme, with a prevalence of 83% (n = 5) in the neoplasm priority lists. Other main research themes, such as ‘Health care system’ and ‘Health condition’, were reflected in 67% (n = 4) of the neoplasm priority lists. The average number of main research themes included in the priority lists on neoplasms was 4.50 and ranged from 2 to 6. ‘Treatment’ was the only main research theme that was mentioned in more than four research priorities assigned in two priority lists.

Five studies prioritized mental and behavioural disorders (F00‐F99).^41^, ^42^, ^44^, ^46^, ^51^ The most prevalent main research themes in the priority lists on mental disorders were ‘Treatment’, ‘Patients’ and ‘HCPs’, and each of these research themes was addressed in 80% of these lists (n = 4). The average number of main research themes included in the priority lists of the individuals affected by mental disorders was 4.60, and the range was 3 to 6. More than four research priorities related to ‘Treatment’ and ‘Patients’ were each addressed in one priority list.

Research priorities regarding the health condition of the genitourinary system (N00 ‐ N99) were developed in four studies.^27^, ^36^, ^38^, ^47^ In 100% of these priority lists, the research theme ‘Patients’ (n = 4) was addressed as the most prevalent main research theme, followed by ‘Treatment’ (n = 3) and ‘Health condition’ (n = 3), each of which was included in 75% of the lists. The average number of main research themes included in the priority lists on the health condition of the genitourinary system was 3.75, and the number of different main research themes ranged from 3 to 5 per list. One JLA priority list comprised more than four research priorities on the main research themes ‘Treatment’ and ‘Health condition’.

## 4 DISCUSSION

We analysed 34 studies in which research priorities were developed with the substantial involvement of patients and caregivers who were directly or indirectly affected by various health conditions. The most prevalent research themes in the investigated priority lists refer to the ‘Treatment’ of the respective health condition, the consequences of the condition and the potential influence of the ‘Patients’ and research in expanding the knowledge about the ‘Health condition’. ‘Treatment’ was one of the three most common research themes in the priority lists of all the differentiated methodological approaches and health conditions.

The priority lists that were developed with or were oriented towards the JLA method are thematically more focused on treatment‐related aspects and cover a smaller range of different themes. Past versions of the JLA guidebook specifically indicate a focus on treatment as they suggest, to prioritize ‘treatment uncertainties’.^53^ With the revisions of the guidebook, this focus has been loosened^12^ and newer versions focus on ‘evidence uncertainties’ which do not need to be assigned to the treatment theme.^13^ ‘Patients’ and ‘Health care system’ were the most prevalent research themes in these lists, thereby highlighting the significance of research on the health‐care system (eg quality of health services) and the patients’ roles in dealing with the health condition (eg through self‐management). These research themes may be underrepresented in JLA‐oriented studies due to the past focus on treatment.

Research priorities on pharmacotherapy are included in ‘Medication’ and comprise 7.55% (n = 25) of the investigated research priorities. With regard to the paper written by Crowe and colleagues,^16^ the present review confirms the mismatch between actual research that clearly focuses on pharmacotherapy and the priorities of the affected individuals.

The underlying health conditions in the lists are very heterogeneous in various aspects. There are relatively rare diseases, such as dystrophic epidermolysis bullosa,^20^ and more common diseases, such as depression^41^ and cancer.^48^ The health conditions also differ in terms of severity, affected areas of life and possible treatment options. Moreover, the level of abstraction varies, as cancer and mental illness were examined specifically and in general. Accordingly, research priorities vary depending on the underlying health condition. For example, the research theme ‘Patients’ includes self‐management issues, and all priority lists related to genitourinary health conditions comprise this research theme. The research priorities of individuals affected by neoplasms are more focused on treatment. Among the priority lists on mental disorders, the research themes ‘Treatment’, ‘Patients’ and ‘HCPs’ are equally prevalent. Compared to other health conditions, the HCP theme is prominent: it is included in 80% of the priority lists for mental disorders, while it can be found in 0 to 33% of the lists focusing on other health conditions.

While the oldest paper included in this review was published in 2005, half of the studies were published since 2016. This finding indicates an increasing body of literature on the development of research priorities involving patients and caregivers. Stewart and colleagues already identified this tendency in their review based on papers published up to 2008.^9^

The JLA and its approach play a pivotal role in the methodology for developing research priorities together with affected individuals. This idea is reflected in the finding that approximately two‐thirds of the included studies were methodologically oriented to the JLA. The importance of the JLA method is also reflected in Yoshida's systematic review of methods used for setting research priorities with and without patient engagement. In this review, the JLA approach was the third most frequently used method.^14^

The JLA is an initiative developed by the United Kingdom (UK) and supported by its government. Accordingly, 12 of the 17 included JLA PSPs were conducted in the UK, 4 were conducted in Canada, and one was conducted in Spain. Of the studies that adopted the JLA method, two were conducted in Canada, one was cross‐nationally conducted in the UK and Ireland, and one study was conducted in each of the following counties: the UK, Germany and Australia. These findings show that the JLA approach is being increasingly recognized internationally. This conclusion is especially true for the JLA‐oriented studies that do not have a formal affiliation with the organization. An enabler for the implementation in the UK and Canada may be the commitment of both governments to support public involvement in their respective health research systems.^54^ The international cooperation and involvement of a JLA supervisor entail increased costs affiliated with an official JLA PSP outside the UK that need to be compensated by research funding programmes. In addition, the difference in language can be a barrier for the adoption of the JLA approach in non‐English‐speaking countries.

### 4.1 Limitations

Our selection criteria excluded studies without substantial involvement of affected individuals. For some health conditions, the symptoms (eg severe cognitive disabilities) limit the patients’ ability to participate in the research priority setting process. For example, a JLA PSP on dementia^55^ was excluded from this review because too few affected persons were involved.

We attempted to adequately map and compare the relevance of research themes across different methods and health conditions and therefore decided to only analyse up to 10 research priorities per study. If the published lists included more than 10 research priorities, the first 10 were included in the analysis. This number is also suggested by the JLA. Moreover, some priority lists included research priorities subsuming different aspects (eg ‘What are the cause, prevention and treatment of itching in dialysis patients?’).^27^ For such interlaced research priorities, we defined the coding rule to consider only the first aspect of each research priority. The coding rule is intended to ensure that research priorities from the different studies are given equal weight. As a consequence, the results may underestimate the variety of research themes especially within priority lists. Decisions in these cases were made by discussions between all three authors.

For the prioritization studies, no specific quality assessment tool is available. The use of a multitude of different instruments would not have been appropriate given the level of methodological heterogeneity. Quality assurance was therefore based on the selection criteria, which also addressed methodological aspects and their documentation. In addition, only pertinent literature databases were searched, and all included articles were published in peer‐reviewed journals.

## 5 CONCLUSIONS

The present review indicates that the relevance of patient involvement in the process of identifying research priorities is increasing. The results provide a comprehensive overview of overarching research themes in research priorities for health research, which were identified and prioritized with the substantial involvement of the affected individuals. The most prevalent research themes relate to research on the ‘Treatment’, ‘Patients’ and ‘Health condition’. In addition to these research themes, a broad range of diverse thematic areas is represented in the investigated priorities. The breadth of the content varies in the included papers depending on the method used and the underlying health problem. The research themes presented in the results can provide guidance to funding bodies.

On the one hand, the review indicates there is variation in priority lists, so disease‐specific approaches are needed. On the other hand, the context should be taken into account, and inter‐country comparisons may further identify international differences. Finally, in order to encourage patient‐centred health research, identified research priorities should be considered and integrated into research activities. Strategies monitoring the uptake of research priorities and reporting their impact should be strengthened.

## CONFLICT OF INTEREST

The authors have no conflicts of interest to disclose.

## Supporting information

Supplementary Material

## Data Availability

The data that support the findings of this study are available from the corresponding author upon reasonable request.
