# Supplementary material for: Priorities of patients, caregivers and health‐care professionals for health research – A systematic review
Source: Health Expect. 2020 Jul 9;23(5):992–1006. doi: 10.1111/hex.13090 (PMC7696132; doi:10.1111/hex.13090)
Supplement: Supplementary file 1 — Supplementary Material [file HEX-23-992-s001.docx]

**Appendices**

**Appendix 1: PRISMA 2009 Checklist**

**Appendix 2: Search strategy**

**Appendix 3: Coding rules**

**Appendix 4: Theme definitions**

**Appendix 1: PRISMA 2009 Checklist**

| **Section/topic** | **#** | **Checklist item** | **Reported on page #** |
| --- | --- | --- | --- |
| **TITLE** | | |  |
| Title | 1 | Identify the report as a systematic review, meta-analysis, or both. | 1 |
| **ABSTRACT** | | |  |
| Structured summary | 2 | Provide a structured summary including, as applicable: background; objectives; data sources; study eligibility criteria, participants, and interventions; study appraisal and synthesis methods; results; limitations; conclusions and implications of key findings; systematic review registration number. | 1-2 |
| **INTRODUCTION** | | |  |
| Rationale | 3 | Describe the rationale for the review in the context of what is already known. | 3-4 |
| Objectives | 4 | Provide an explicit statement of questions being addressed with reference to participants, interventions, comparisons, outcomes, and study design (PICOS). | 4 |
| **METHODS** | | |  |
| Protocol and registration | 5 | Indicate if a review protocol exists, if and where it can be accessed (eg Web address), and, if available, provide registration information including registration number. | 4 |
| Eligibility criteria | 6 | Specify study characteristics (eg PICOS, length of follow-up) and report characteristics (eg years considered, language, publication status) used as criteria for eligibility, giving rationale. | 5 / 17 |
| Information sources | 7 | Describe all information sources (eg databases with dates of coverage, contact with study authors to identify additional studies) in the search and date last searched. | 4 |
| Search | 8 | Present full electronic search strategy for at least one database, including any limits used, such that it could be repeated. | 28 |
| Study selection | 9 | State the process for selecting studies (ie screening, eligibility, included in systematic review, and, if applicable, included in the meta-analysis). | 5 |
| Data collection process | 10 | Describe method of data extraction from reports (eg piloted forms, independently, in duplicate) and any processes for obtaining and confirming data from investigators. | 5 |
| Data items | 11 | List and define all variables for which data were sought (eg PICOS, funding sources) and any assumptions and simplifications made. | 5 |
| Risk of bias in individual studies | 12 | Describe methods used for assessing risk of bias of individual studies (including specification of whether this was done at the study or outcome level), and how this information is to be used in any data synthesis. | 5 |
| Summary measures | 13 | State the principal summary measures (eg risk ratio, difference in means). | 5 |
| Synthesis of results | 14 | Describe the methods of handling data and combining results of studies, if done, including measures of consistency (eg I^2^) for each meta-analysis. | 5 |

Page 1 of 2

| **Section/topic** | **#** | **Checklist item** | **Reported on page #** |
| --- | --- | --- | --- |
| Risk of bias across studies | 15 | Specify any assessment of risk of bias that may affect the cumulative evidence (eg publication bias, selective reporting within studies). | 5 |
| Additional analyses | 16 | Describe methods of additional analyses (eg sensitivity or subgroup analyses, meta-regression), if done, indicating which were pre-specified. | N/A |
| **RESULTS** | | |  |
| Study selection | 17 | Give numbers of studies screened, assessed for eligibility, and included in the review, with reasons for exclusions at each stage, ideally with a flow diagram. | 6 / 30 |
| Study characteristics | 18 | For each study, present characteristics for which data were extracted (eg study size, PICOS, follow-up period) and provide the citations. | 18-20 |
| Risk of bias within studies | 19 | Present data on risk of bias of each study and, if available, any outcome level assessment (see item 12). | 5 |
| Results of individual studies | 20 | For all outcomes considered (benefits or harms), present, for each study: (a) simple summary data for each intervention group (b) effect estimates and confidence intervals, ideally with a forest plot. | 21-23 |
| Synthesis of results | 21 | Present results of each meta-analysis done, including confidence intervals and measures of consistency. | 6-10 |
| Risk of bias across studies | 22 | Present results of any assessment of risk of bias across studies (see Item 15). | 5 |
| Additional analysis | 23 | Give results of additional analyses, if done (eg sensitivity or subgroup analyses, meta-regression [see Item 16]). | N/A |
| **DISCUSSION** | | |  |
| Summary of evidence | 24 | Summarize the main findings including the strength of evidence for each main outcome; consider their relevance to key groups (eg health-care providers, users, and policymakers). | 11-12 |
| Limitations | 25 | Discuss limitations at study and outcome level (eg risk of bias), and at review level (eg incomplete retrieval of identified research, reporting bias). | 12-13 |
| Conclusions | 26 | Provide a general interpretation of the results in the context of other evidence, and implications for future research. | 13 |
| **FUNDING** | | |  |
| Funding | 27 | Describe sources of funding for the systematic review and other support (eg supply of data); role of funders for the systematic review. | Title page |

*From:* Moher D, Liberati A, Tetzlaff J, Altman DG, The PRISMA Group (2009). Preferred Reporting Items for Systematic Reviews and Meta-Analyses: The PRISMA Statement. PLoS Med 6(7): e1000097. doi:10.1371/journal.pmed1000097

**Appendix 2: Search strategy**

| **Databases** | **Syntax** | **Key words** |
| --- | --- | --- |
| **PubMed** | ((((patients[TIAB] OR carers[TIAB] OR "service users"[TIAB] OR clients[TIAB] OR consumers[TIAB] OR lay[TIAB])) AND priorit*[TIAB]) AND research[TIAB]) OR ("James Lind Alliance"[TIAB] OR "James Lind Initiative"[TIAB]) | patients OR carers OR "service users" OR clients OR consumers OR lay  AND  priorit*  AND  research  OR  "James Lind Alliance" OR "James Lind Initiative" |
| **Scopus** | ( TITLE-ABS-KEY ( patients OR carers OR "service users" OR clients OR consumers OR lay ) AND TITLE-ABS-KEY ( ( priorit* W/2 research ) ) OR TITLE-ABS-KEY ( "James Lind Alliance" OR "James Lind Initiative" ) ) | patients OR carers OR "service users" OR clients OR consumers OR lay  AND  ( priorit* W/2 research )  OR  "James Lind Alliance" OR "James Lind Initiative" |
| **Ovid - PsycINFO** | (((patients or carers or 'service users' or clients or consumers or lay) and (priorit* adj2 research)) or ('James Lind Alliance' or 'James Lind Initiative')).mp. | patients OR carers OR 'service users' OR clients OR consumers OR lay  AND  (priorit* adj2 research)  OR  'James Lind Alliance' or 'James Lind Initiative' |

**Appendix 3: Coding rules**

**Coding rules**

1. Coding follows the question ´What does the intended research focus on?´
2. Each research priority will be assigned to one research theme.
3. Research priorities that comprise multiple interlaced aspects will be coded according to the aspect (research theme) mentioned first.
4. The first 10 research themes of each priority list will be coded. This also applies if multiple research priorities share a ranking position. Then, the first ten research priorities will be considered according to the listing.

**Appendix 4: Theme descriptions**

| **Theme** / *Subtheme* | Description | Examples |
| --- | --- | --- |
| **Prevention**  *Prevention of disease*  *Identify and manage at-risk groups* | The theme is subdivided into the two subthemes ´Prevention of disease´ and ´Identify and manages at risk groups´.  ‘Prevention of disease’ covers themes, which focus on research priorities to develop ways of preventing the onset of the disease.  ‘Identify and manage at risk groups’ contains research priorities regarding screening measures, which aim to identify target groups for preventive treatment options. | ‘What are the most effective new interventions and treatments to prevent the development and progression of kidney disease?’ ^38^  ‘Can a simpler, more accurate and convenient screening test be developed for GDM?’ ^37^ |
| **Diagnosis** | Research priorities, which aim to allow a (more precise or earlier) ‘diagnosis’ of the disease and related symptoms as well as questions on relevant factors in this regard are aggregated in this subtheme. | ‘Identification and validation of biomarkers that may be used for the detection of kidney cancer.’ ^58^ |
| **Treatment**  *Monitoring*  *Patient involvement in treatment*  *Development*  *Further medical therapy*  *Further therapy*  *Psychotherapy*  *Medication*  *Effective treatment*  *Personalized and patient-centred care* | The main theme ‘treatment’ is based on the nine subthemes ‘monitoring’, ‘patient involvement in treatment’, ‘development’, ‘further medical therapy’, ‘further therapy’, ‘psychotherapy’, ‘medication’, ‘effective treatment’ and ‘personalized and patient-centered care’.  ‘Monitoring’ includes research priorities that relate to monitoring measures for vital signs (eg home blood pressure monitoring or 24-hour ambulatory blood pressure monitoring) and monitoring as optimization of routine measurements and follow-ups.  The subtheme ‘patient involvement in treatment’ includes research priorities regarding the possibilities of actively involving patients in their own health care or health services, for example through Shared Decision Making.  ‘Development’ includes research priorities that focus on innovative, pioneering research. These include research priorities on the general development of new effective treatments and research priorities to clarify the role of a certain therapy option in treatment (eg tissue engineering).  ‘Further medical therapy’ includes all research priorities dealing with different treatments that must be performed by medical staff (eg endoscopic treatment, surgical procedure). Research priorities are about proof of efficacy (eg by insulin pump therapy), long-term results (eg by syndactyly surgery), comparative effectiveness research (eg best surgical procedure) and benefits (eg by pleurectomy) of a certain treatment on different diseases.  ‘Further therapy’ includes all research priorities dealing with different treatments that are not performed by medical staff (eg by an occupational therapist). Research priorities are about proof of efficacy (eg rest/elevation during an episode of cellulitis), comparative effectiveness research (eg best speech and language therapy) of a certain treatment on different diseases as well as rehabilitation strategies to improve health outcomes. There are also research priorities about this theme, which focus on natural or alternative therapies and guidelines for a therapy.  ‘Psychotherapy’ includes research priorities in which psychological interventions and their effectiveness and the combination from psychological therapy with other therapy forms are addressed.  ‘Medication’ includes all general research priorities that belong to the subject medication. This includes research priorities on different forms of therapy (eg hormone therapy, chemotherapy), forms of application (eg oral treatment), as well as research priorities on drug combinations (eg antibiotics), side-effects (eg less side-effects, long- term complications) and drug interactions (eg mutual interaction). In addition, research priorities about proof of efficacy, ask how effective a drug is, and comparative effectiveness research (eg most effective drug, best medication) of a drug are included. Research priorities on side-effects of drugs are assigned to the theme medications.  ‘Effective treatment’ includes all general research priorities that ask for the best therapy/method/ strategy, treatment recommendations or effectiveness of a therapy on diseases. This theme also includes research priorities that are looking for the best way to improve understanding or manage or prevent a disease.  ‘Personalized and patient-centered care’ includes research priorities on personalized treatments and patient-centred care. Personalized and patient-centred care is related to the patients´ individual needs and preferences in developing and implementing health services and characteristics of individual patients or patient groups, also needs of ethnic groups and culturally therapy alternatives. Research priorities also ask about personalization due to different types of disease and their optimal conditions for a treatment. Also included are research priorities that relate to predict benefits of treatment or treatment response. | ‘Is it possible to constantly and accurately monitor blood sugar levels, in people with Type 1 diabetes, with a discrete device (non-invasive or invasive)?’ ^32^  ‘involving patients in therapeutic decisions’ ^43^  ‘Innovative, pioneering research for improves wound healing’ ^50^  ‘What is the best surgical procedure to perform in treating HS, e.g. incision and drainage, local excision, wide excision?’ ^31^  ‘Which speech and language therapy treatments work best for aphasia?’ ^39^  ‘What psychological interventions would improve the psychological health for transition between kidney stages?’ ^36^  ‘Which antibiotic combinations and dosing plans should be used for Cystic Fibrosis exacerbations and should antibiotic combinations be rotated?’ ^25^  ‘What are the most effective treatments currently available for advanced EC and what key molecular pathways should be targeted when developing new treatments?’ ^21^  ‘Is the duration, dose and method of administration of antibiotics needed to treat cellulitis related to patient characteristics (eg patients with diabetes who are overweight or have swelling of the limb may require a higher dose/duration)?’ ^23^ |
| **(informal) carers** | Research priorities in this theme deal with carers need for education and further emotional support. | ‘How can carers and others be helped to communicate with someone with aphasia?’ ^39^ |
| **HCPs** | Research priorities that focus on HCPs regarding their attitudes, roles, education, communication and effectiveness. | ‘How can healthcare professionals be best supported to accurately diagnose and manage cellulitis and to advise their patients in how to prevent relapses?’ ^23^ |
| **Patients**  *Patient behaviour*  *Participation in social/ work life*  *Psychosocial consequences*  *Patient education*  *Information needs* | The theme ‘patients’ is divided into five subthemes. These reflect information for patients (‘Information needs’, ‘Patient education’) and patient behaviour as well as the consequences of an illness for the patients (‘Psychosocial consequences’, ‘Participation in social/work life’).  ‘Patient behavior’: This theme is about what patients themselves can do to manage their illness. Research priorities focusing on roles as well as effects of lifestyle changes as well as self-management and self-help strategies have been linked to this theme.  ‘Participation in social/ work life’: Research priorities that ask for strategies to reduce impairments in participation (eg travelling, working) are subsumed in this theme.  ‘Psychosocial consequences’: This theme deals with the psychological burden of a disease. This includes coping with adverse feelings as emotional adaptation.  ‘Patient education’: Research priorities concern the design of patient education programmes as well as the mode of delivery.  ‘Information needs’: This theme subsumes research priorities that are related to the information needs of patients. There is some information that is not adequately assessable or understandable for patients, especially with cognitive impairments. | ‘What makes self management successful for some people with Type 1 diabetes, and not others?’ ^32^  ‘What strategies help patients maintain work while on HD?’ ^36^  ‘What are the emotional and mental health impacts of miscarriage in the short term and long term for the mother and the partner?’ ^30^  ‘What are optimal educational tools, strategies, and technologies to improve patient motivation and health behavior change for hypertension?’ ^29^  'Find out what people with learning disabilities understand about being healthy and keeping themselves healthy.’ ^42^ |
| *Public awareness and education* | Themes, which indicate a need for research to raise public awareness towards the patients’ situations, are comprised in this theme. Specifically, research priorities ask how awareness and educational campaigns can be made more effective in order to improve the situation of those affected and to decrease stigmatization. | ‘What ways of raising public awareness about EC are the most effective and cost effective?’ ^21^ |
| **Health-care System**  *(Availability &) Accessibility*  *Coordination*  *Costs*  *Quality of Services* | The theme ‘health care system’ comprises themes which address research on health services, on the situation of people affected in the health-care system and ways for optimization in this regard. Within this theme, the subthemes Accessibility, Coordination, Costs and Quality of Services can be found, which are specified as follows  ‘(Availability &) Accessibility’: Related research priorities focus on the accessibility of health services used or needed by the specific patient group. Moreover, research priorities ask for the extent of which affected individuals have equal access to health care or for possible barriers in this regard.  ‘Coordination’: Research priorities affiliated to this subtheme address an optimization of organizational aspects and the coordination of health-care processes within the system.  ‘Costs’: Research priorities of this subtheme deal with research on financial and societal costs caused by the disease. In addition, research priorities regarding the financing of reimbursements and health services needed by those affected are comprised in this subtheme.  ‘Quality of Services’: Research priorities of this subtheme ask to investigate the effectiveness of health services. In this context, the relevance of specific aspects such as cultural factors or institutional framework conditions for the quality of services is questioned as well as needs of patients which are unmet by present health services. | ‘How do we ensure that patients with CKD have equitable access to care (eg nephrologists, allied health clinics) irrespective of location of residence or socio-economic status?’ ^38^  ‘How can patient information (about women with GDM) be better shared across health providers and the healthcare system to improve coordination and provision of care?’ ^37^  ‘Evaluation of the impact of differences in regional funding and access to treatment on patient outcomes for kidney cancer.’ ^58^  ‘How much does management in reference centers help patients with DEB (in terms of quality of life, avoiding complications and disability, cost-effectiveness)?’ ^20^ |
| **Health Condition**  *Causes of Disease*  *Comorbidity*  *Course of Disease*  *More Information on epidemiological data* | Contents assigned to the theme ‘health condition’ comprise research priorities, which aim to enhance knowledge on the specific aspects of the health condition. Within this theme the subthemes Causes of Disease, Comorbidity, and Course of Disease can be found, which are specified as follows:  ‘Causes of Disease’: Research priorities from this subtheme ask for research on risk factors and causes for the onset of the disease or associated symptoms.  ‘Comorbidity’: These research priorities focus on comorbidities of the specific disease. These research priorities ask for research on the relationship between disease and comorbidity as well as for possibilities to manage and prevent comorbidities.  ‘Course of Disease’: Research priorities in this subtheme refer to factors that influence the course of a disease and might cause an exacerbation or improvement. In addition, research priorities on factors or diagnostic procedures, which allow predicting the course of disease, are comprised in this subtheme.  ‘More Information on epidemiological data’: This subtheme contains only one research priority that aims to map epidemiological data on burn accidents | ‘Do demographic, dietary or other factors (including past histories of GDM) play a role in developing GDM, and can GDM be prevented?’ ^37^  ‘More knowledge on the relation between asthma/ COPD and other diseases (co-morbidity))’ ^49^  ‘Which factors are useful in determining the prognosis (disease progression) of HS?’ ^20^  ‘Mapping epidemiological data on burn accidents in the Netherlands’ ^50^ |
